# Supplementary figures and images for: Selective impact of ALK and MELK inhibition on ERα stability and cell proliferation in cell lines representing distinct molecular phenotypes of breast cancer
Source: Sci Rep. 2024 Apr 8;14:8200. doi: 10.1038/s41598-024-59001-x (PMC11001865; doi:10.1038/s41598-024-59001-x)

## Slide 1
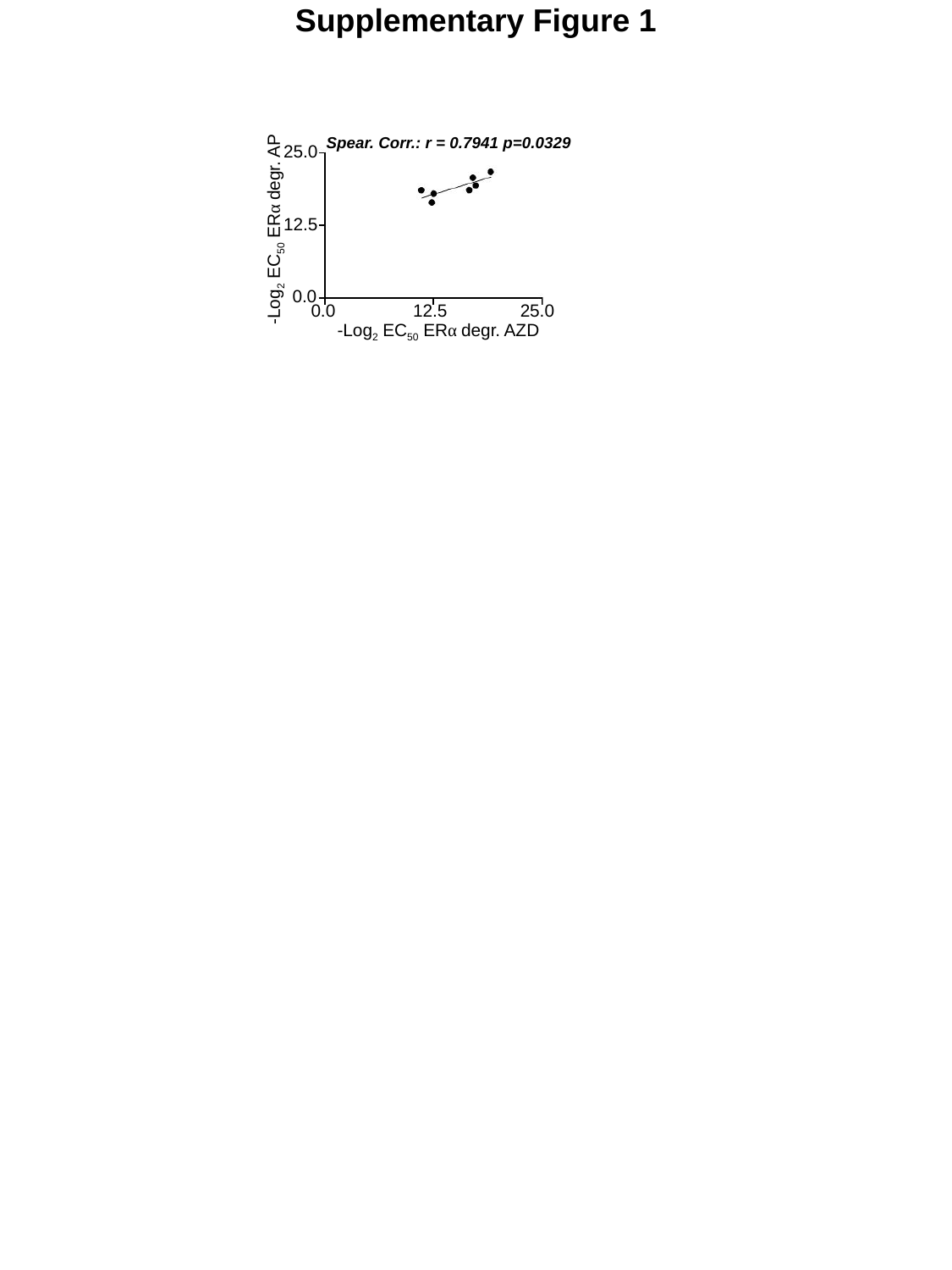

Supplementary Figure 1
Spear. Corr.: r = 0.7941 p=0.0329
25.0
12.5
-Log2 EC50 ERα degr. AP
0.0
0.0
12.5
25.0
-Log2 EC50 ERα degr. AZD

Supplement: Supplementary file 6 — Supplementary Figure 1. [file 41598_2024_59001_MOESM6_ESM.pptx]

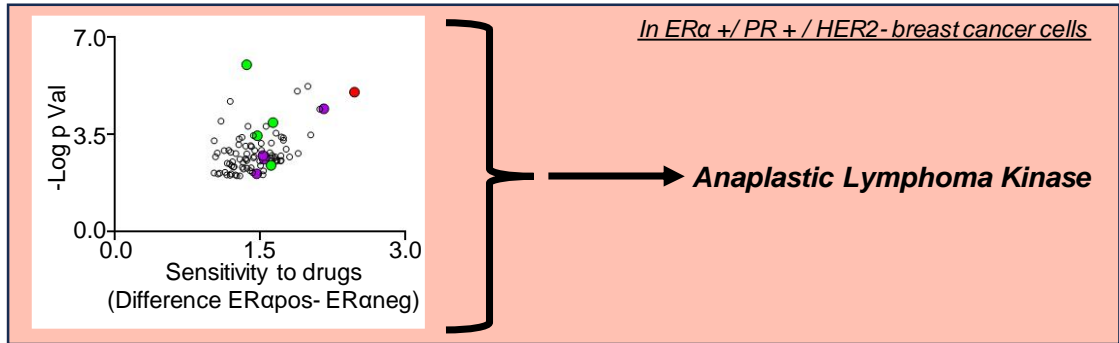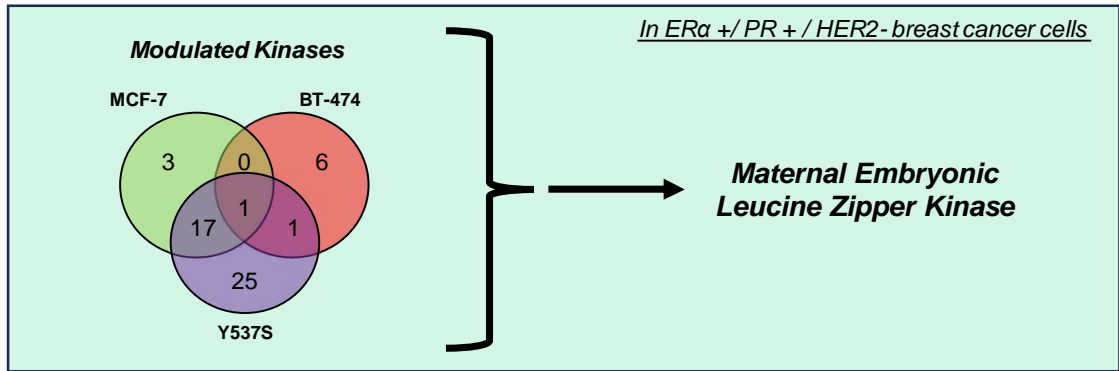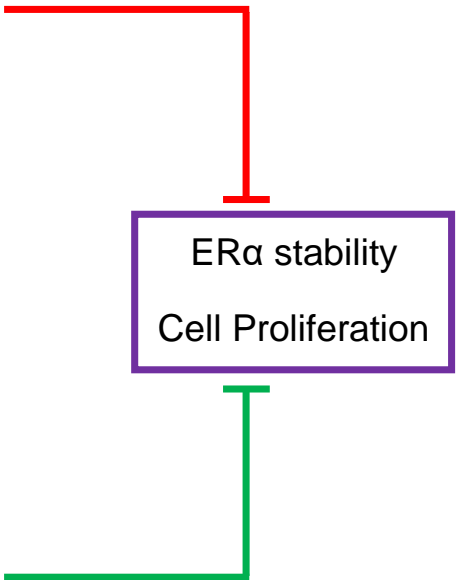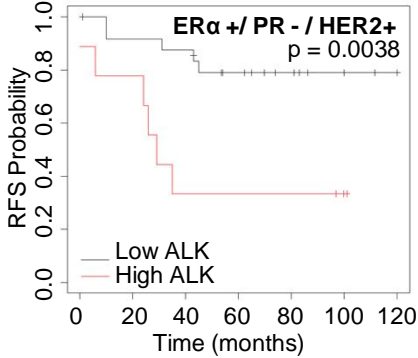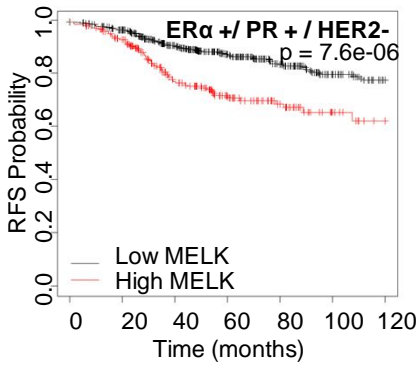

Supplement: Supplementary file 10 — Supplementary Information 10. [file 41598_2024_59001_MOESM10_ESM.pdf]
