# Supplementary material for: Selective impact of ALK and MELK inhibition on ERα stability and cell proliferation in cell lines representing distinct molecular phenotypes of breast cancer
Source: Sci Rep. 2024 Apr 8;14:8200. doi: 10.1038/s41598-024-59001-x (PMC11001865; doi:10.1038/s41598-024-59001-x)
Supplement: Supplementary file 7 — Supplementary Figure 2. [file 41598_2024_59001_MOESM7_ESM.pptx]

## Slide 1
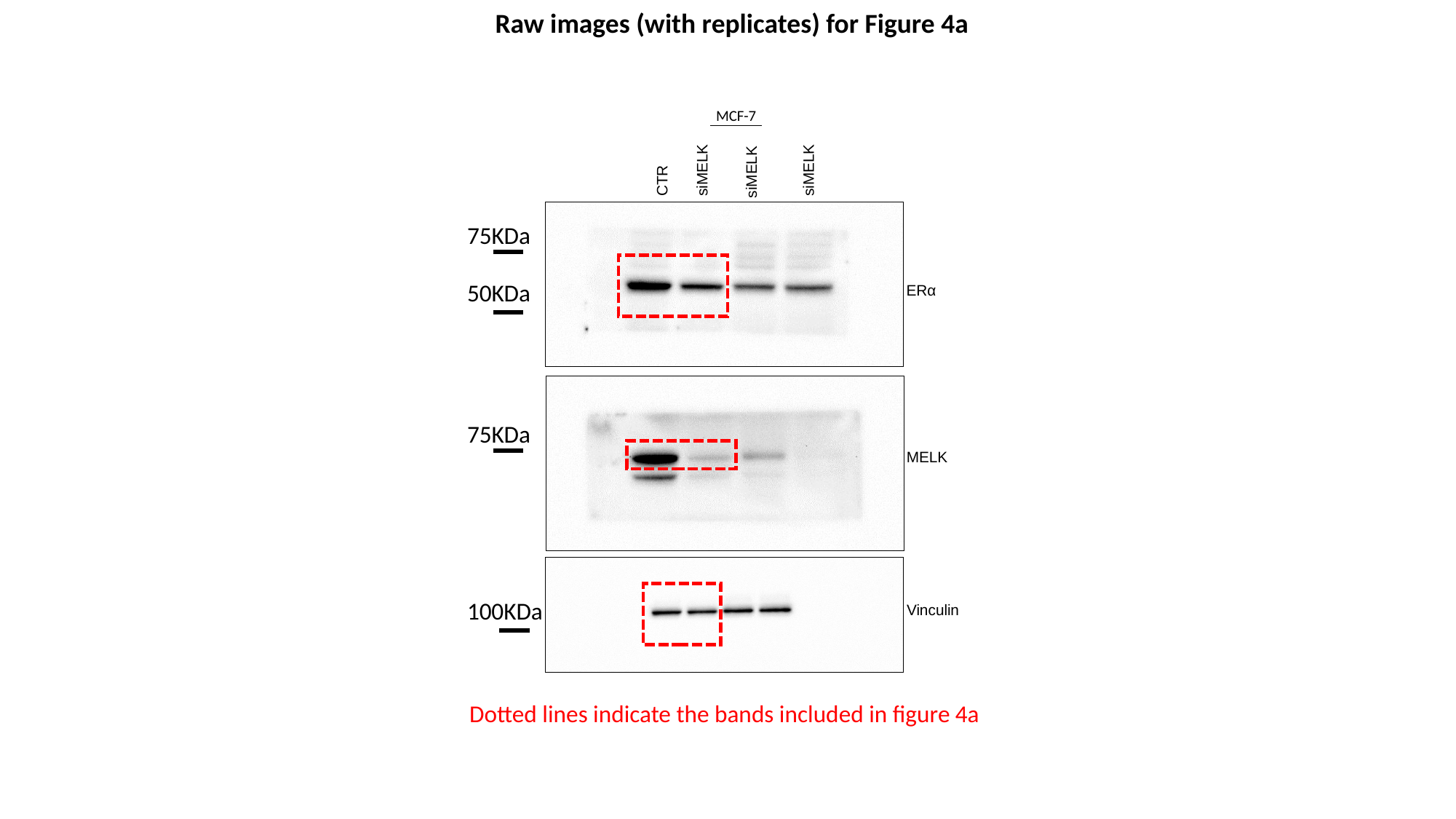

Raw images (with replicates) for Figure 4a
MCF-7
siMELK
siMELK
siMELK
CTR
75KDa
50KDa
ERα
75KDa
MELK
100KDa
Vinculin
Dotted lines indicate the bands included in figure 4a

## Slide 2
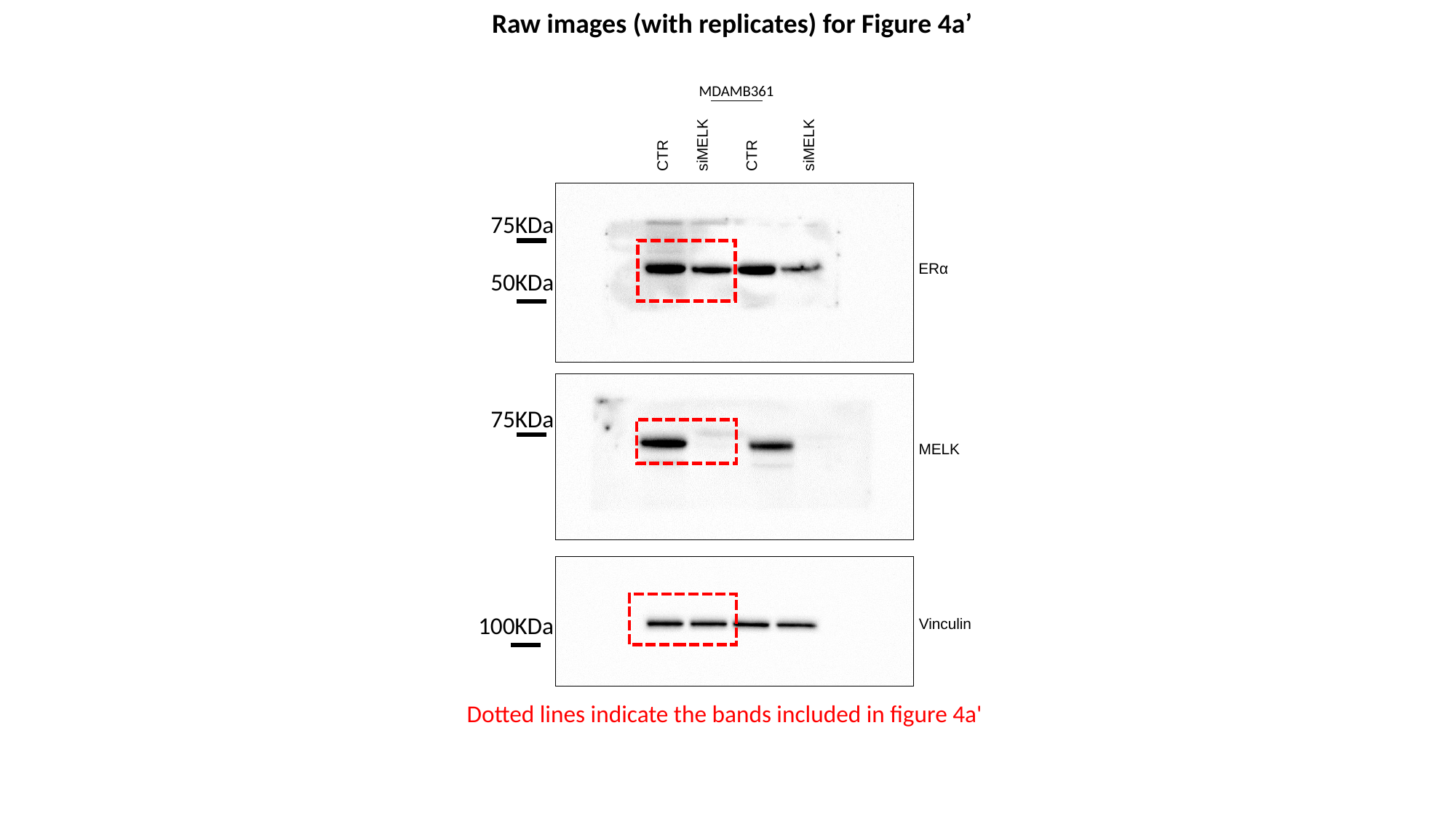

Raw images (with replicates) for Figure 4a’
MDAMB361
siMELK
siMELK
CTR
CTR
75KDa
50KDa
ERα
75KDa
MELK
100KDa
Vinculin
Dotted lines indicate the bands included in figure 4a'

## Slide 3
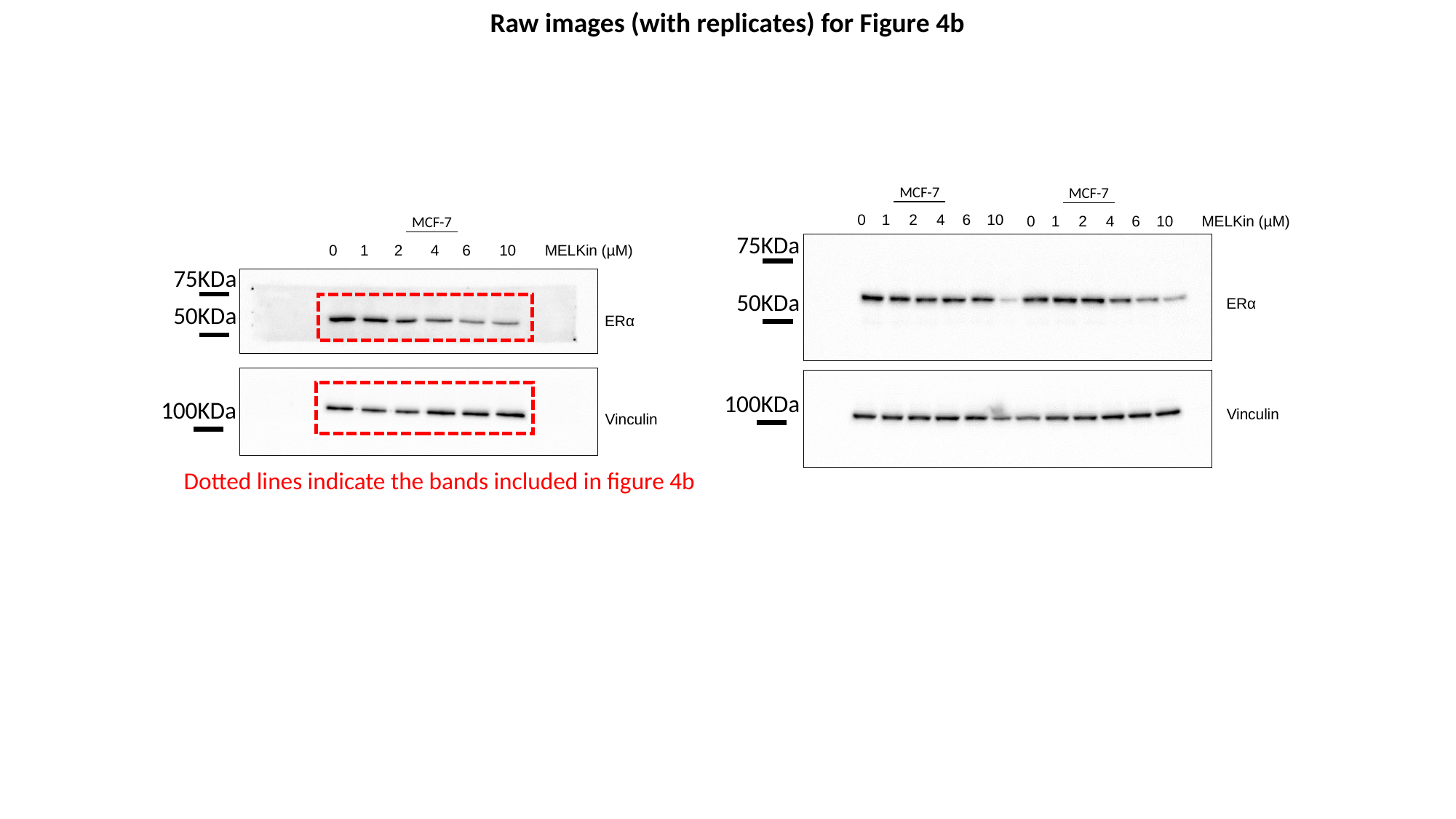

Raw images (with replicates) for Figure 4b
MCF-7
MCF-7
MCF-7
0
1
2
4
6
10
0
1
2
4
6
10
MELKin (µM)
75KDa
50KDa
0
1
2
4
6
10
MELKin (µM)
75KDa
50KDa
ERα
ERα
100KDa
100KDa
Vinculin
Vinculin
Dotted lines indicate the bands included in figure 4b

## Slide 4
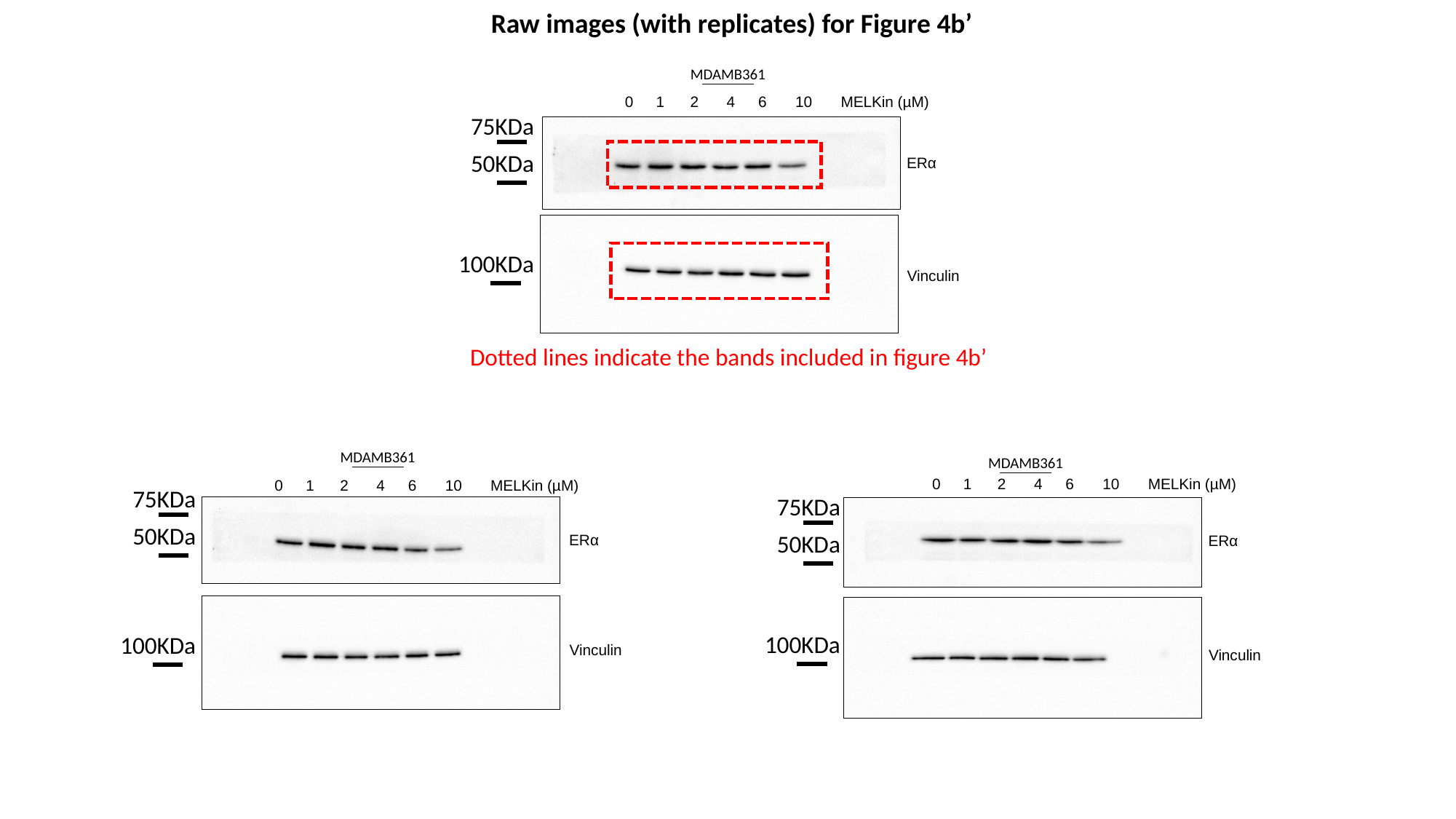

Raw images (with replicates) for Figure 4b’
MDAMB361
0
1
2
4
6
10
MELKin (µM)
75KDa
50KDa
ERα
100KDa
Vinculin
Dotted lines indicate the bands included in figure 4b’
MDAMB361
MDAMB361
0
1
2
4
6
10
MELKin (µM)
0
1
2
4
6
10
MELKin (µM)
75KDa
50KDa
75KDa
50KDa
ERα
ERα
100KDa
100KDa
Vinculin
Vinculin

## Slide 5
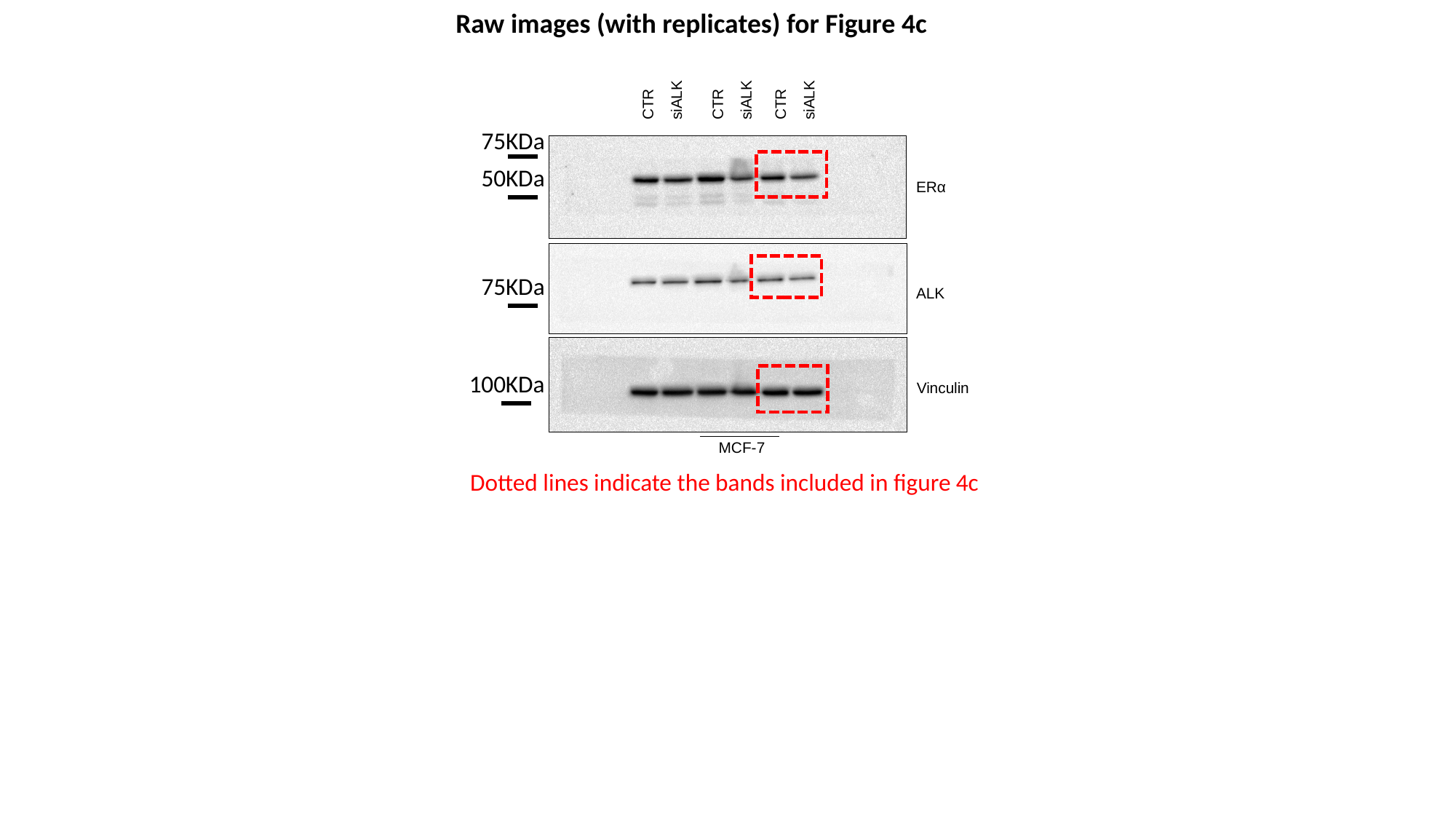

Raw images (with replicates) for Figure 4c
siALK
siALK
siALK
CTR
CTR
CTR
75KDa
50KDa
ERα
75KDa
ALK
100KDa
Vinculin
MCF-7
Dotted lines indicate the bands included in figure 4c

## Slide 6
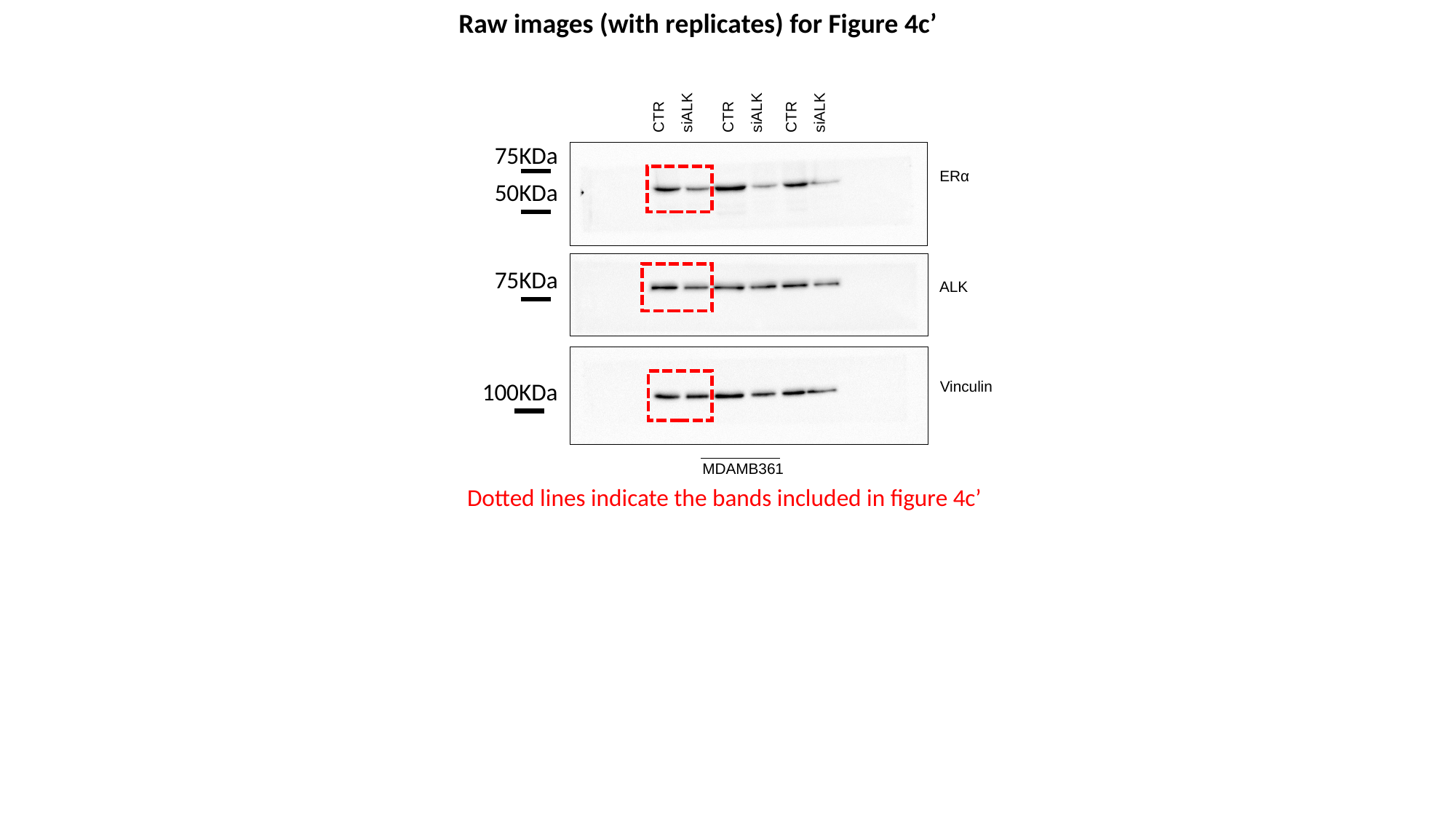

Raw images (with replicates) for Figure 4c’
siALK
siALK
siALK
CTR
CTR
CTR
75KDa
50KDa
ERα
75KDa
ALK
100KDa
Vinculin
MDAMB361
Dotted lines indicate the bands included in figure 4c’

## Slide 7
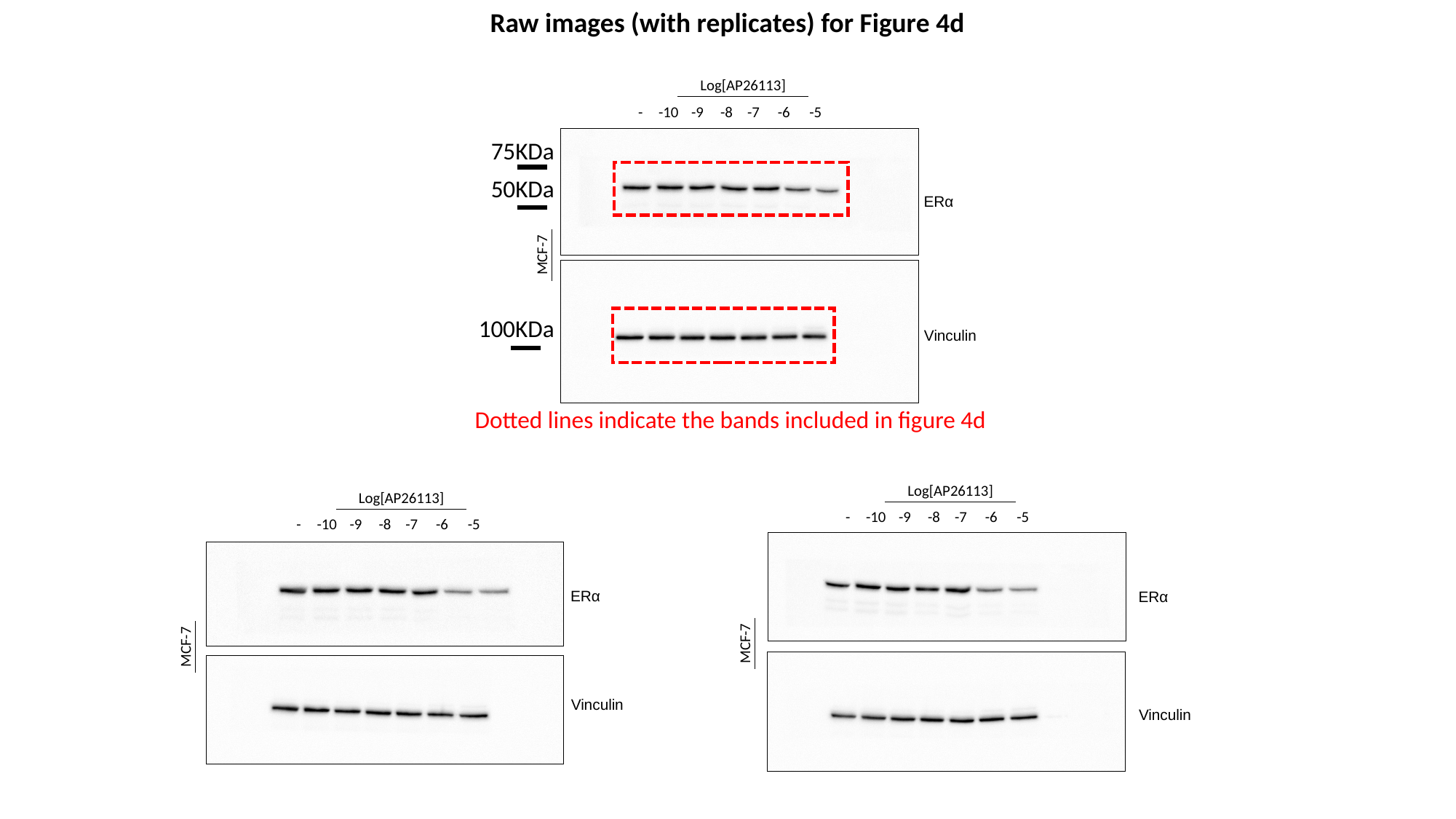

Raw images (with replicates) for Figure 4d
Log[AP26113]
-
-10
-9
-8
-7
-6
-5
75KDa
50KDa
ERα
MCF-7
100KDa
Vinculin
Dotted lines indicate the bands included in figure 4d
Log[AP26113]
-
-10
-9
-8
-7
-6
-5
ERα
MCF-7
Vinculin
Log[AP26113]
-
-10
-9
-8
-7
-6
-5
ERα
MCF-7
Vinculin

## Slide 8
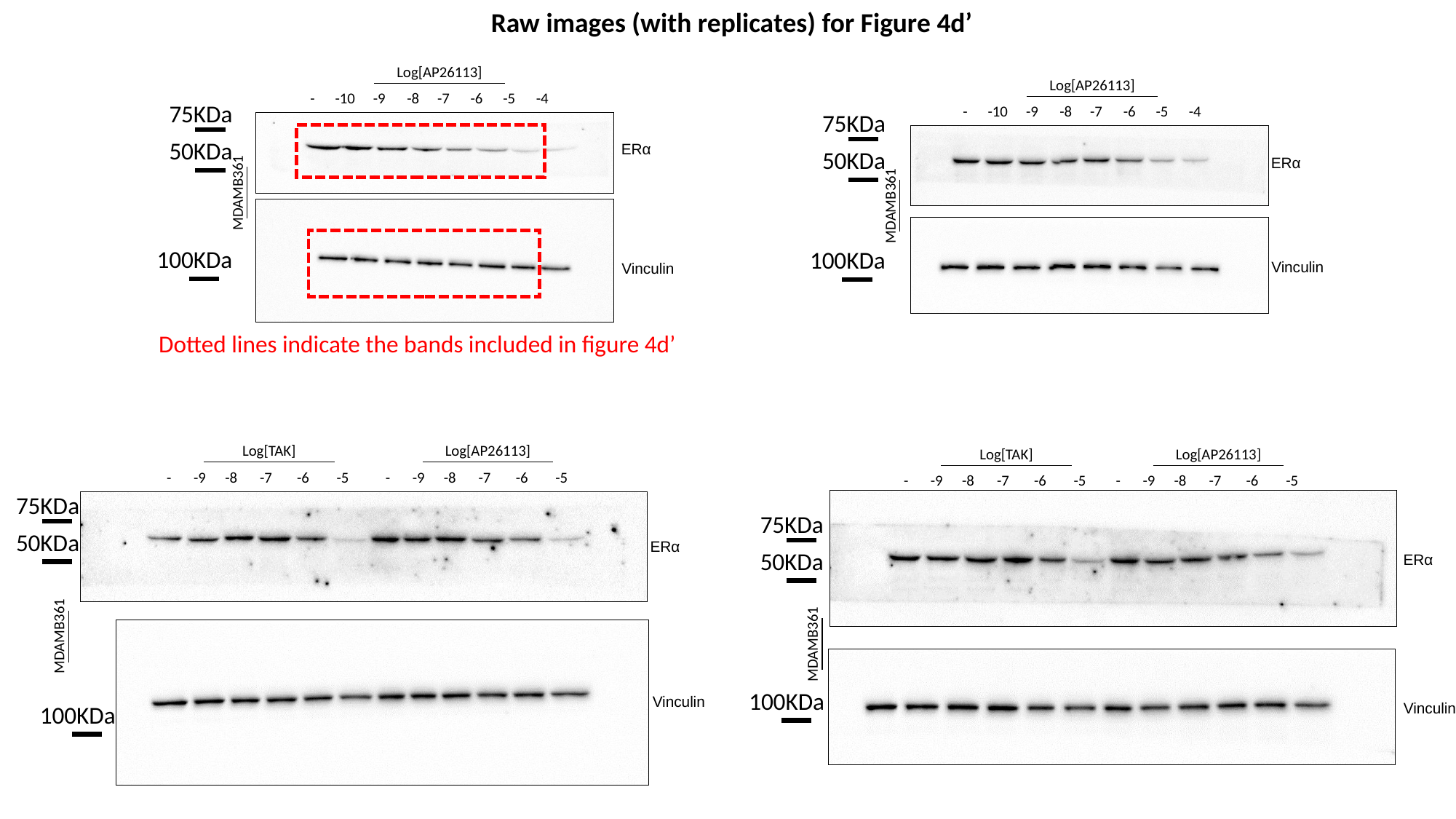

Raw images (with replicates) for Figure 4d’
Log[AP26113]
Log[AP26113]
-
-10
-9
-8
-7
-6
-5
-4
ERα
MDAMB361
Vinculin
-
-10
-9
-8
-7
-6
-5
-4
75KDa
50KDa
75KDa
50KDa
ERα
MDAMB361
100KDa
100KDa
Vinculin
Dotted lines indicate the bands included in figure 4d’
Log[TAK]
Log[AP26113]
-
-9
-8
-7
-6
-5
-
-9
-8
-7
-6
-5
75KDa
50KDa
ERα
MDAMB361
Vinculin
100KDa
Log[TAK]
Log[AP26113]
-
-9
-8
-7
-6
-5
-
-9
-8
-7
-6
-5
75KDa
50KDa
ERα
MDAMB361
100KDa
Vinculin

## Slide 9
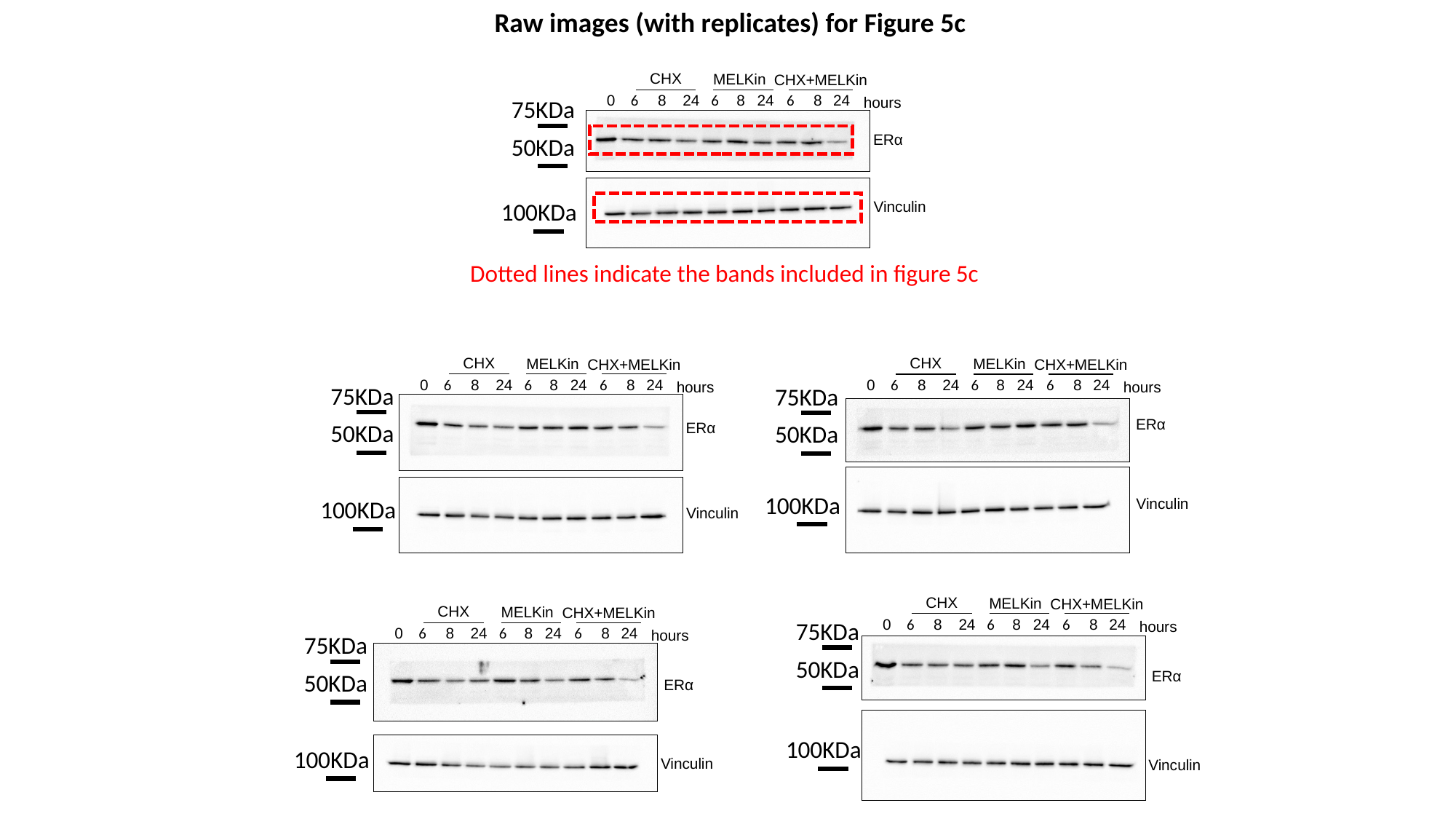

Raw images (with replicates) for Figure 5c
CHX
MELKin
CHX+MELKin
0
6
8
24
6
8
24
6
8
24
hours
75KDa
50KDa
ERα
100KDa
Vinculin
Dotted lines indicate the bands included in figure 5c
CHX
MELKin
CHX+MELKin
0
6
8
24
6
8
24
6
8
24
hours
75KDa
50KDa
ERα
100KDa
Vinculin
CHX
MELKin
CHX+MELKin
0
6
8
24
6
8
24
6
8
24
hours
75KDa
50KDa
ERα
100KDa
Vinculin
CHX
MELKin
CHX+MELKin
0
6
8
24
6
8
24
6
8
24
hours
ERα
Vinculin
75KDa
50KDa
100KDa
CHX
MELKin
CHX+MELKin
0
6
8
24
6
8
24
6
8
24
hours
ERα
Vinculin
75KDa
50KDa
100KDa

## Slide 10
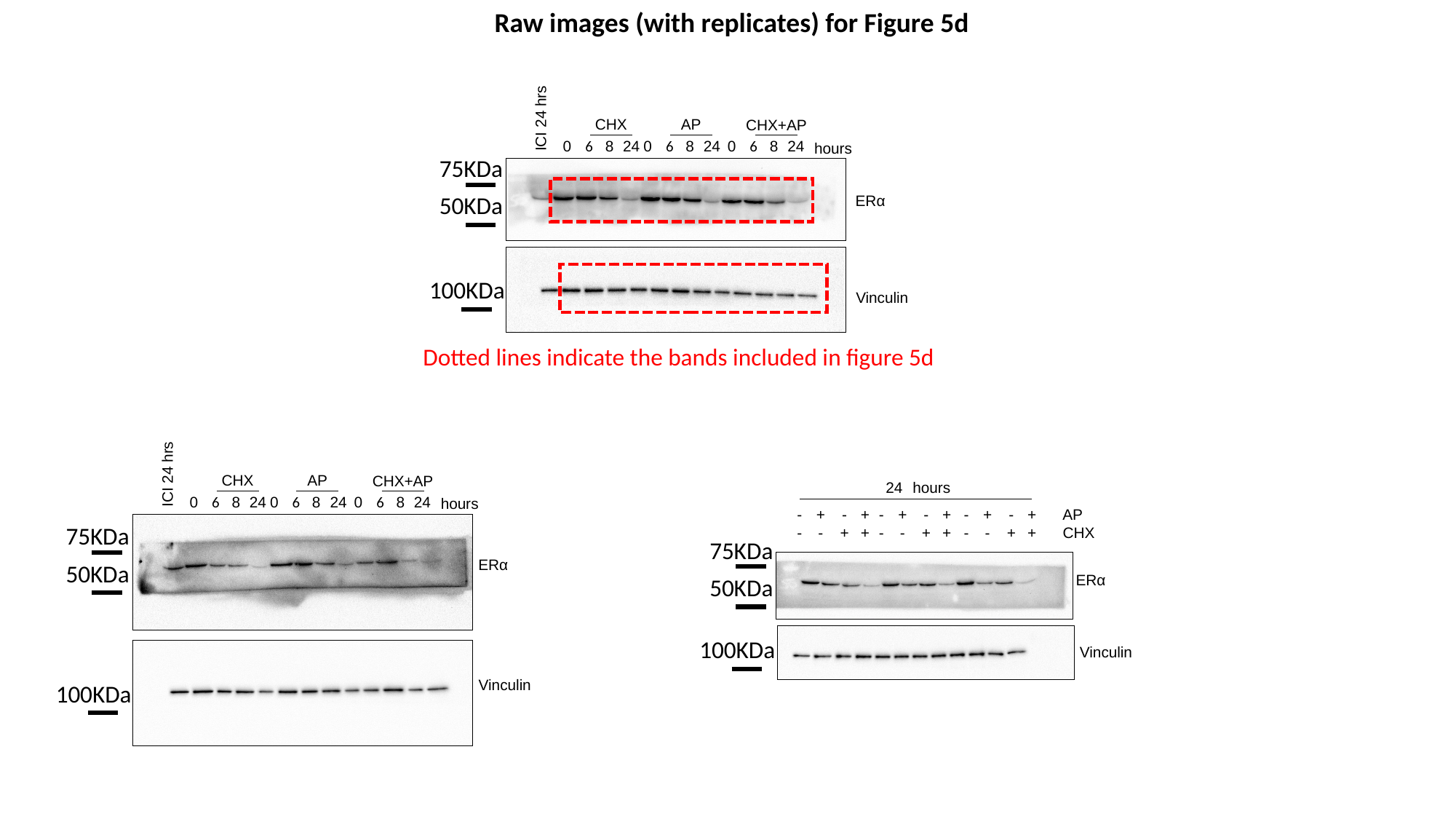

Raw images (with replicates) for Figure 5d
ICI 24 hrs
CHX
AP
CHX+AP
0
6
8
24
0
6
8
24
0
6
8
24
hours
75KDa
50KDa
ERα
100KDa
Vinculin
Dotted lines indicate the bands included in figure 5d
ICI 24 hrs
CHX
AP
CHX+AP
0
6
8
24
0
6
8
24
0
6
8
24
hours
ERα
Vinculin
24
hours
-
+
-
+
-
-
+
+
-
+
-
+
-
-
+
+
-
+
-
+
-
-
+
+
AP
CHX
ERα
Vinculin
75KDa
50KDa
75KDa
50KDa
100KDa
100KDa

## Slide 11
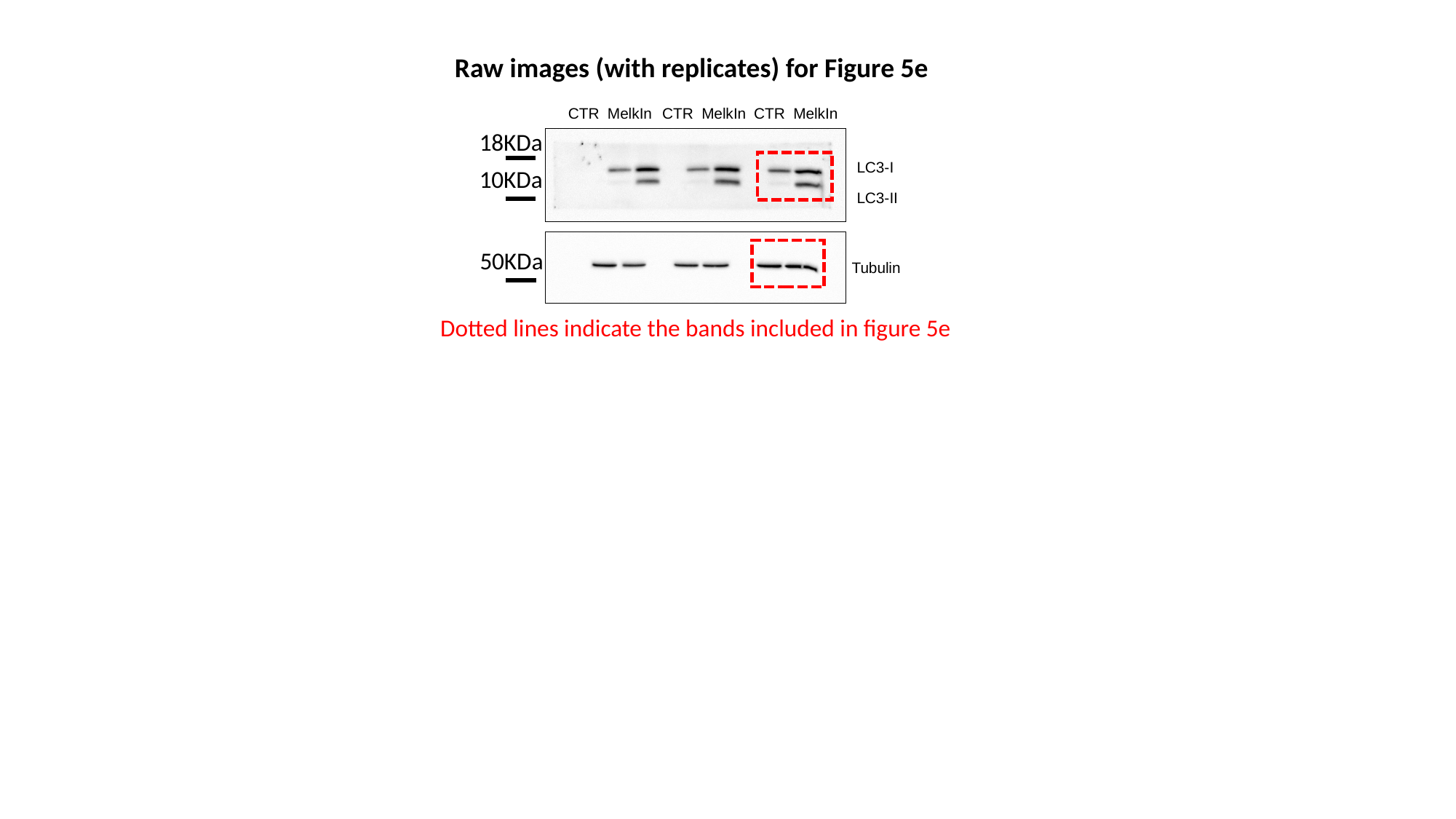

Raw images (with replicates) for Figure 5e
CTR
MelkIn
CTR
MelkIn
CTR
MelkIn
LC3-I
LC3-II
Tubulin
18KDa
10KDa
50KDa
Dotted lines indicate the bands included in figure 5e

## Slide 12
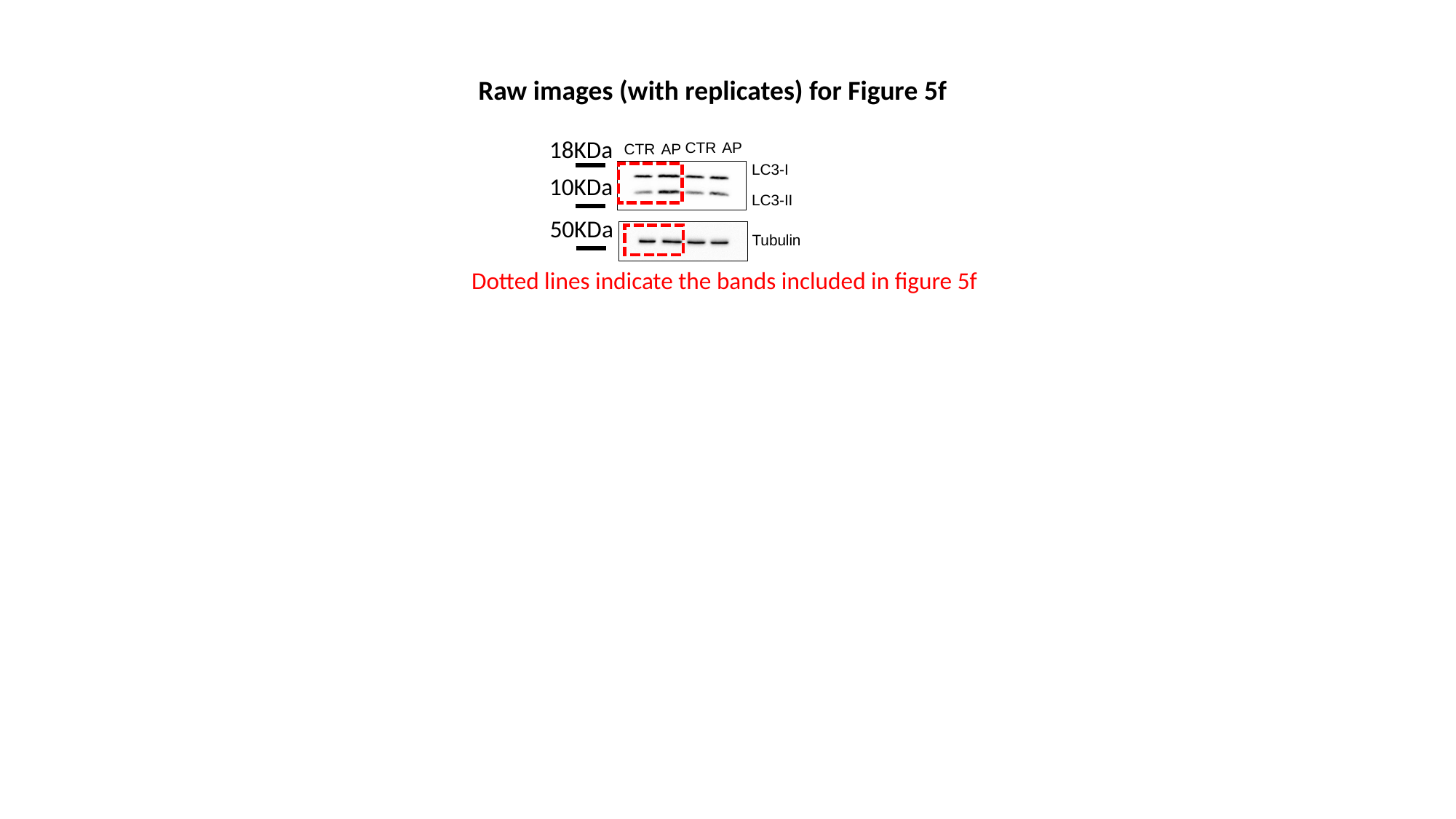

Raw images (with replicates) for Figure 5f
18KDa
10KDa
CTR
AP
CTR
AP
LC3-I
LC3-II
50KDa
Tubulin
Dotted lines indicate the bands included in figure 5f

## Slide 13
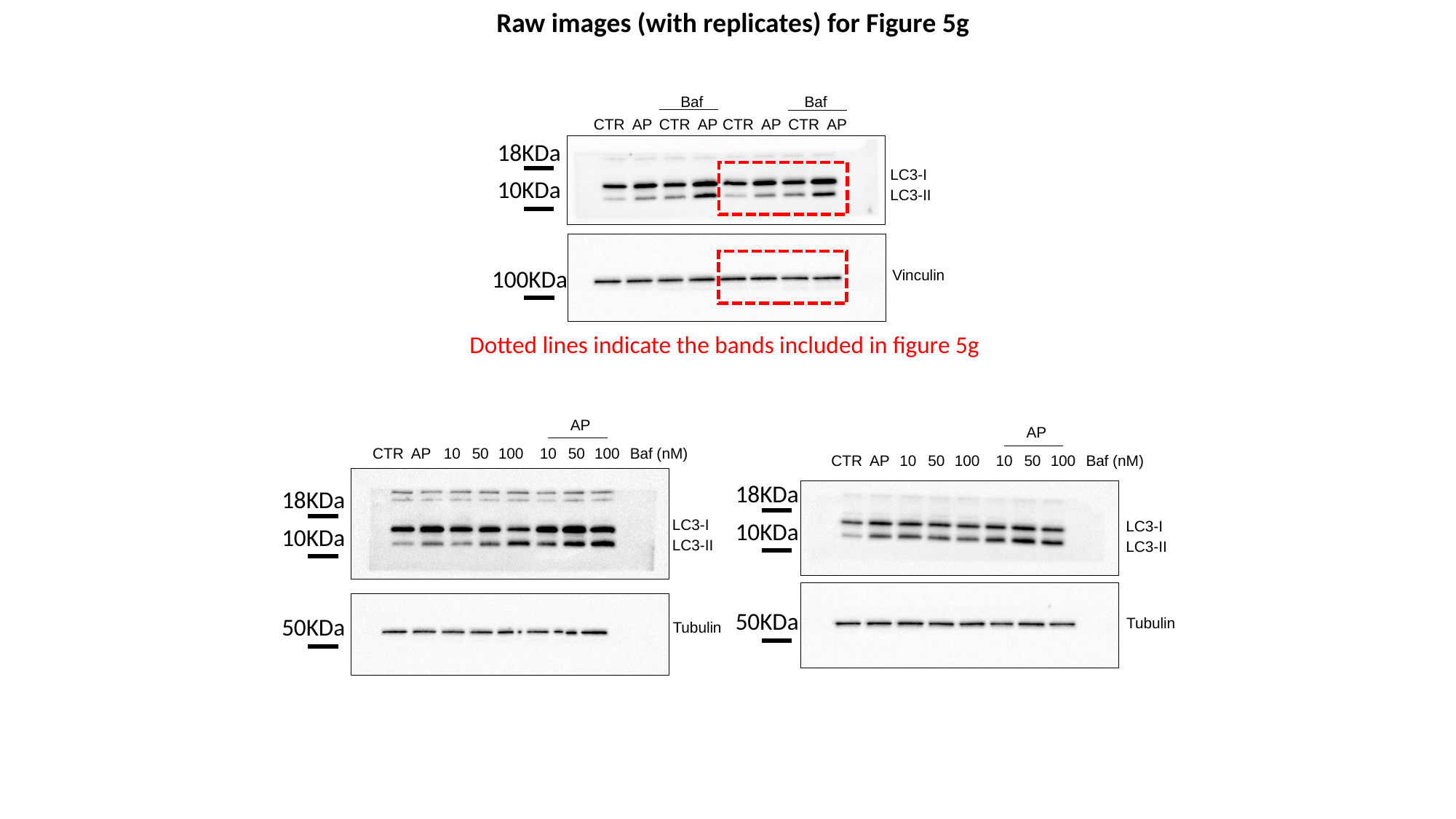

Raw images (with replicates) for Figure 5g
Baf
Baf
CTR
AP
CTR
AP
CTR
AP
CTR
AP
18KDa
10KDa
LC3-I
LC3-II
100KDa
Vinculin
Dotted lines indicate the bands included in figure 5g
AP
CTR
AP
10
50
100
10
50
100
Baf (nM)
18KDa
LC3-I
10KDa
LC3-II
50KDa
Tubulin
AP
CTR
AP
10
50
100
10
50
100
Baf (nM)
LC3-I
LC3-II
Tubulin
18KDa
10KDa
50KDa

## Slide 14
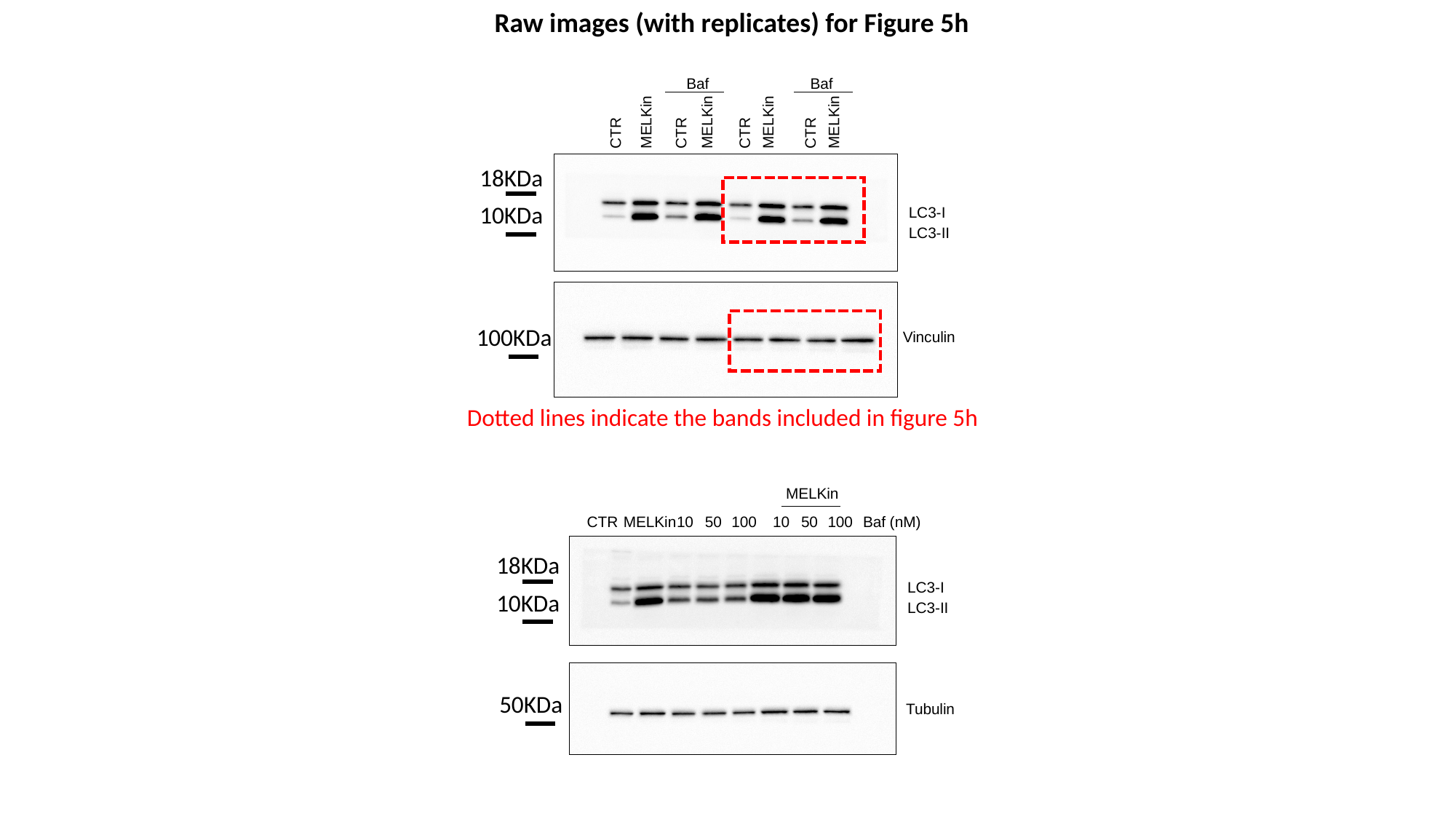

Raw images (with replicates) for Figure 5h
Baf
Baf
MELKin
MELKin
MELKin
MELKin
CTR
CTR
CTR
CTR
18KDa
10KDa
LC3-I
LC3-II
100KDa
Vinculin
Dotted lines indicate the bands included in figure 5h
MELKin
CTR
MELKin
10
50
100
10
50
100
Baf (nM)
LC3-I
LC3-II
Tubulin
18KDa
10KDa
50KDa

## Slide 15
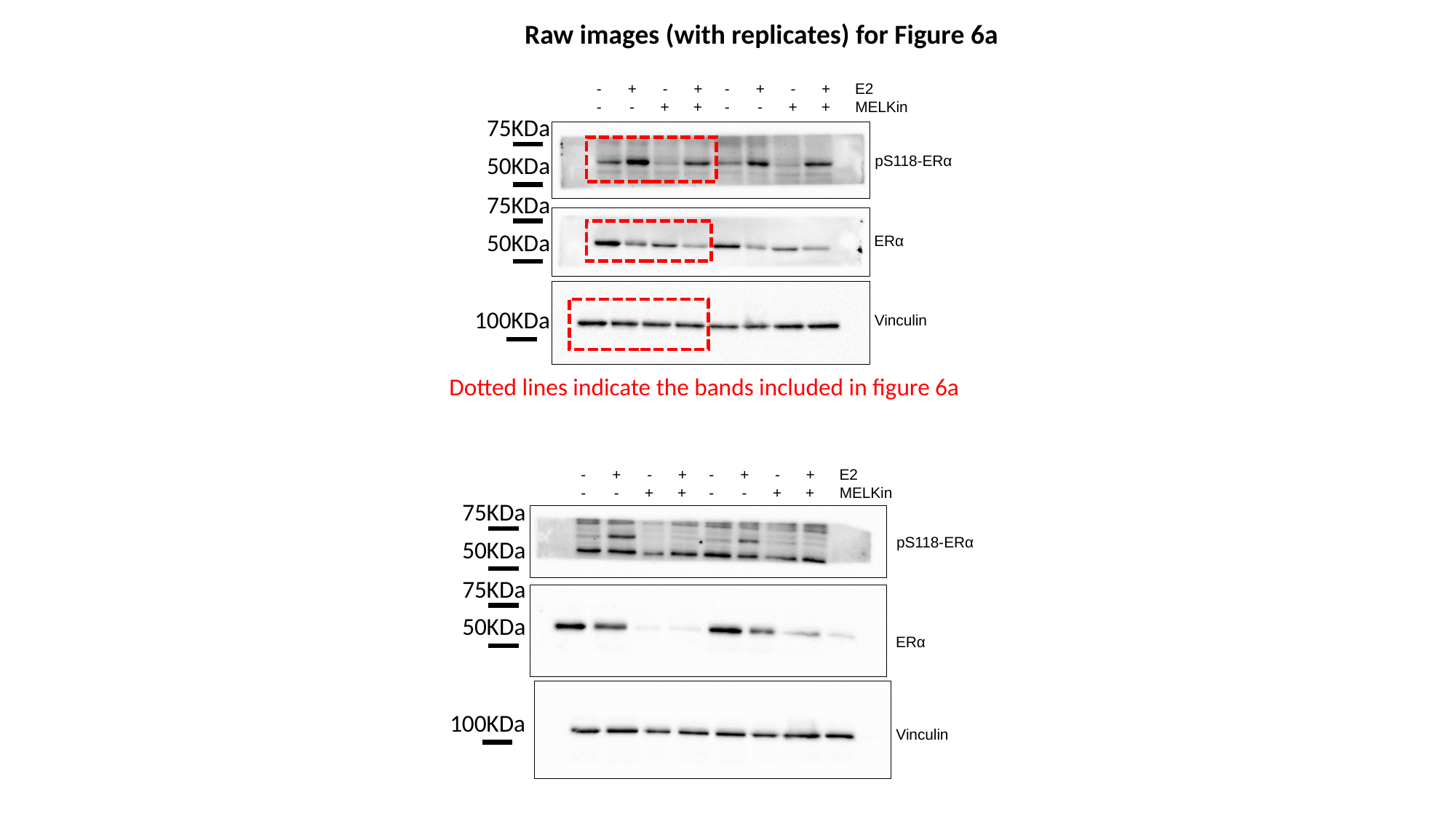

Raw images (with replicates) for Figure 6a
-
+
-
+
-
-
+
+
-
+
-
+
-
-
+
+
E2
MELKin
75KDa
50KDa
pS118-ERα
75KDa
50KDa
ERα
100KDa
Vinculin
Dotted lines indicate the bands included in figure 6a
-
+
-
+
-
-
+
+
-
+
-
+
-
-
+
+
E2
MELKin
ERα
Vinculin
pS118-ERα
75KDa
50KDa
75KDa
50KDa
100KDa

## Slide 16
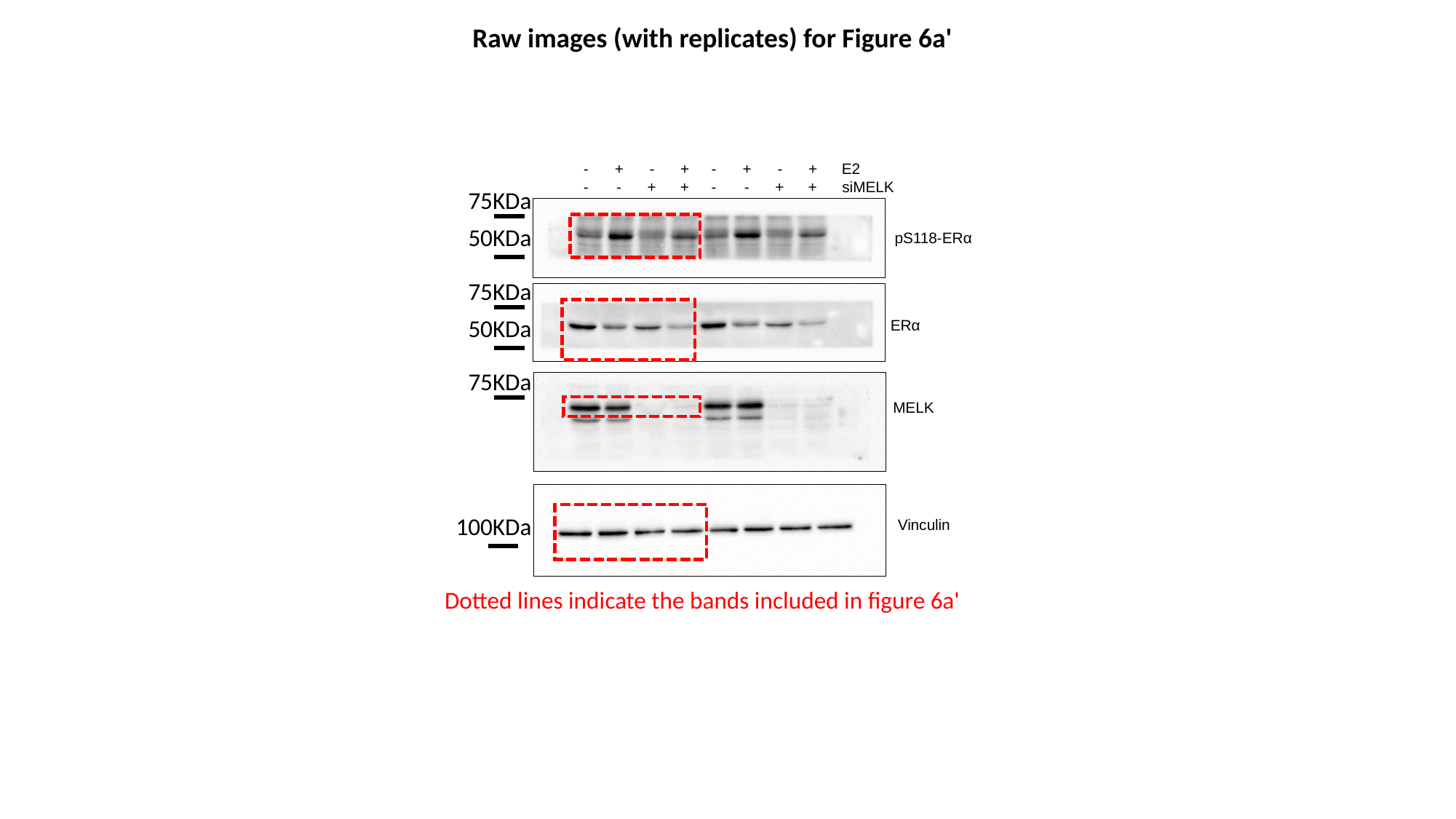

Raw images (with replicates) for Figure 6a'
-
+
-
+
-
-
+
+
-
+
-
+
-
-
+
+
E2
siMELK
pS118-ERα
ERα
MELK
Vinculin
75KDa
50KDa
75KDa
50KDa
75KDa
100KDa
Dotted lines indicate the bands included in figure 6a'

## Slide 17
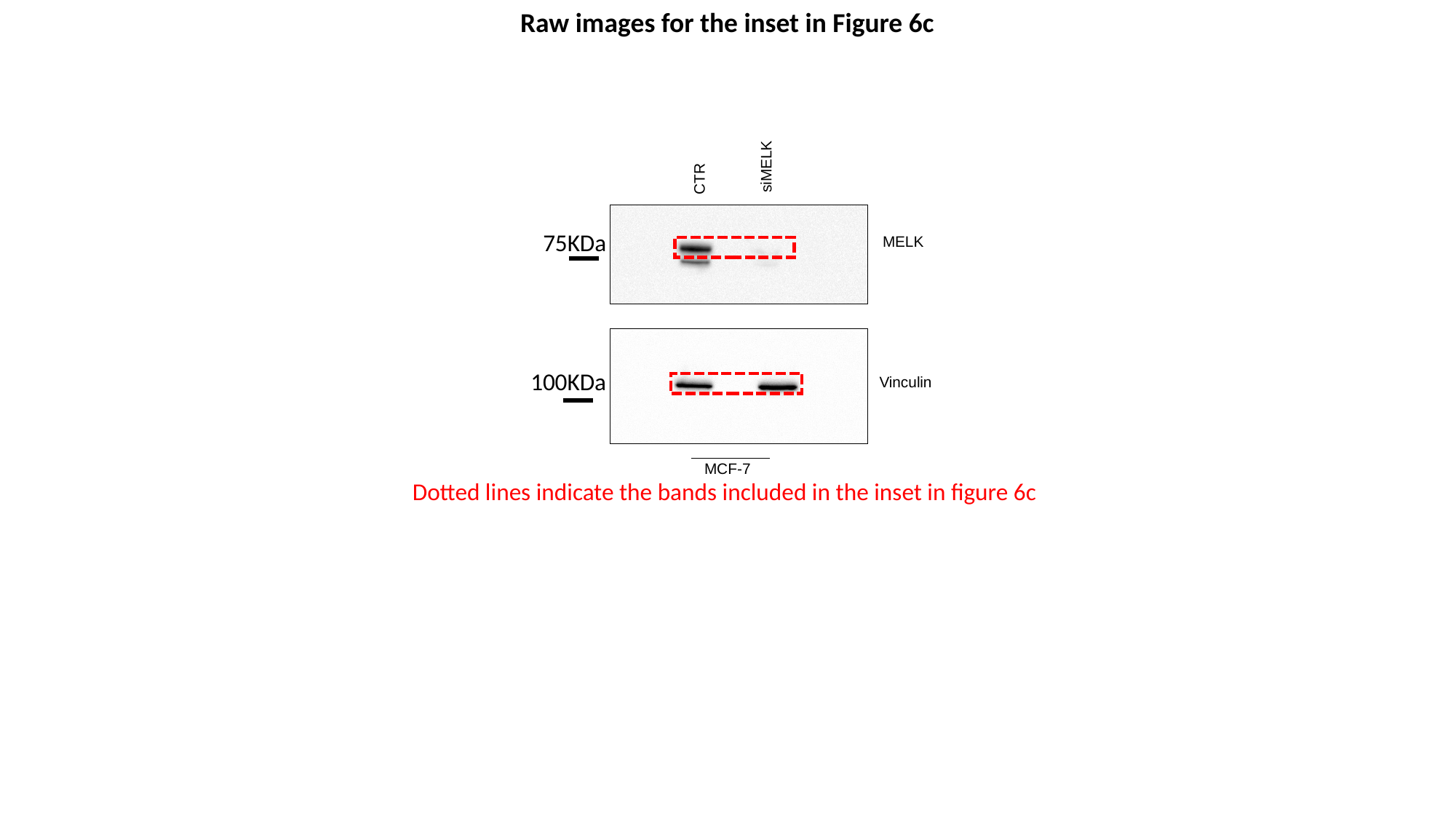

Raw images for the inset in Figure 6c
siMELK
CTR
MELK
Vinculin
MCF-7
75KDa
100KDa
Dotted lines indicate the bands included in the inset in figure 6c

## Slide 18
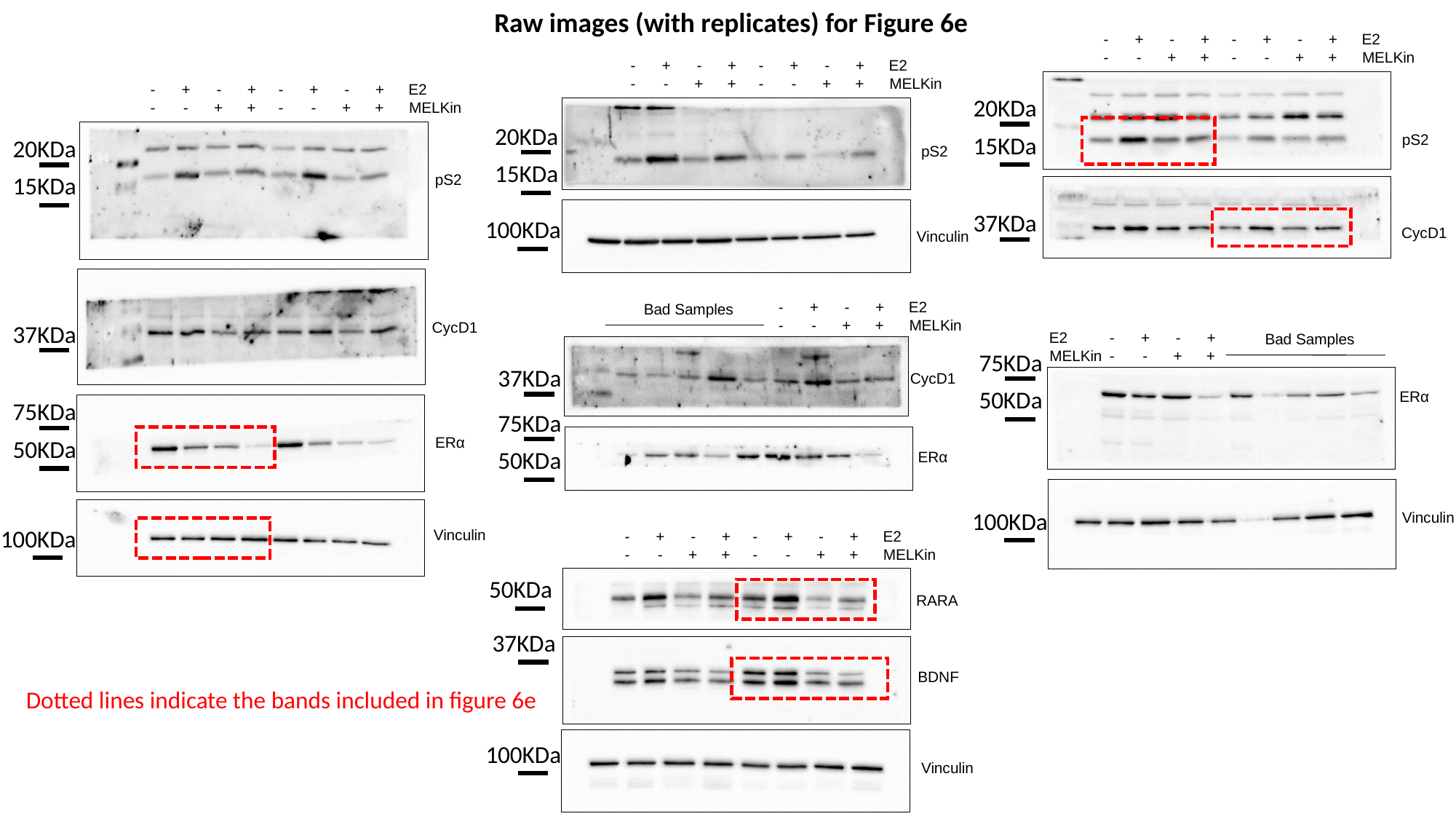

Raw images (with replicates) for Figure 6e
-
+
-
+
-
-
+
+
-
+
-
+
-
-
+
+
E2
MELKin
-
+
-
+
-
-
+
+
-
+
-
+
-
-
+
+
E2
MELKin
20KDa
15KDa
pS2
100KDa
Vinculin
-
+
-
+
-
-
+
+
E2
Bad Samples
MELKin
37KDa
CycD1
75KDa
50KDa
ERα
-
+
-
+
-
-
+
+
-
+
-
+
-
-
+
+
E2
MELKin
RARA
BDNF
Vinculin
50KDa
37KDa
100KDa
-
+
-
+
-
-
+
+
-
+
-
+
-
-
+
+
E2
MELKin
pS2
CycD1
ERα
Vinculin
20KDa
15KDa
pS2
20KDa
15KDa
37KDa
CycD1
37KDa
E2
-
+
-
+
-
-
+
+
Bad Samples
MELKin
75KDa
50KDa
ERα
75KDa
50KDa
100KDa
Vinculin
100KDa
Dotted lines indicate the bands included in figure 6e

## Slide 19
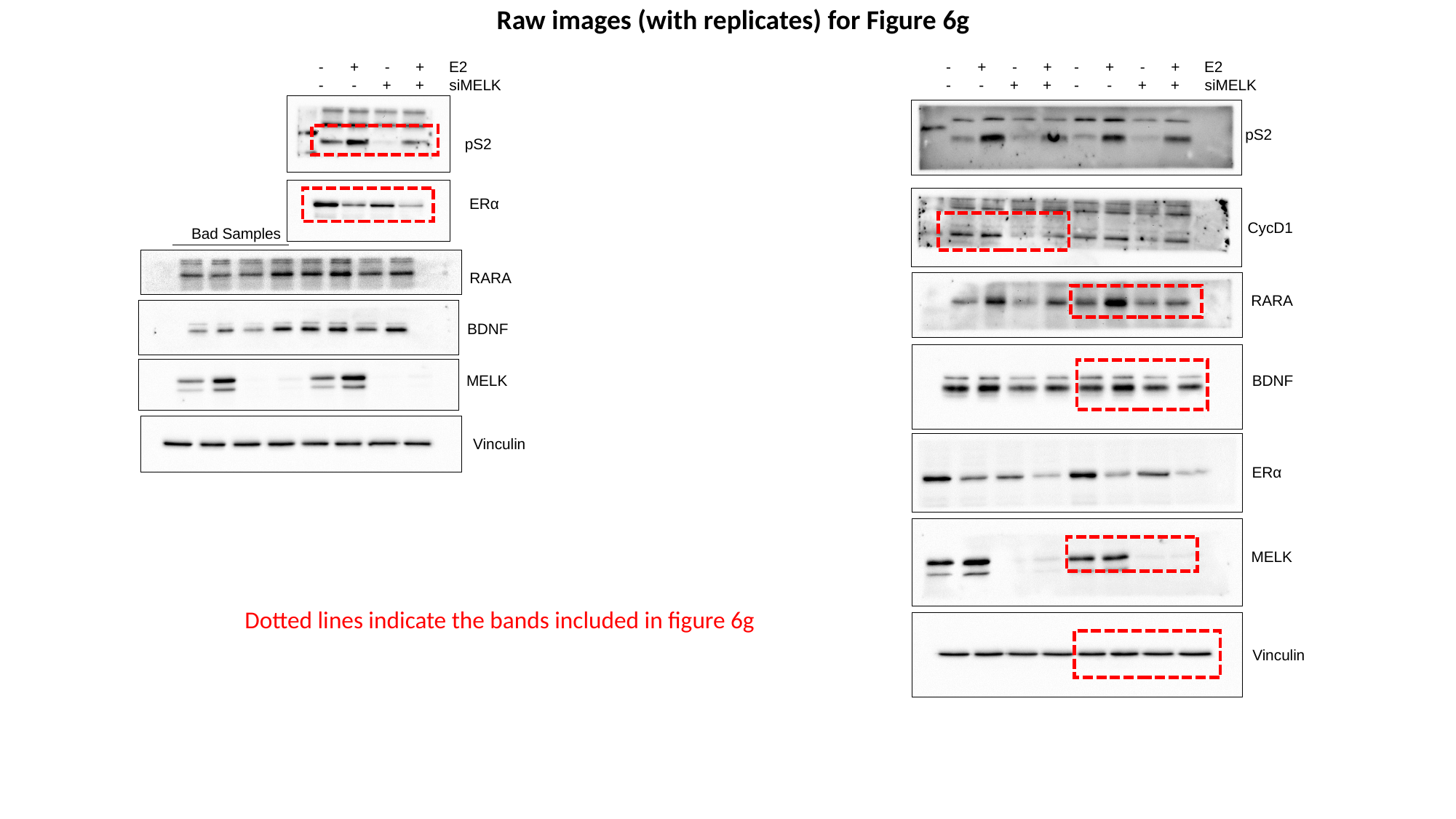

Raw images (with replicates) for Figure 6g
-
+
-
+
-
-
+
+
E2
-
+
-
+
-
-
+
+
-
+
-
+
-
-
+
+
E2
siMELK
siMELK
pS2
pS2
ERα
CycD1
Bad Samples
RARA
RARA
BDNF
BDNF
MELK
Vinculin
ERα
MELK
Dotted lines indicate the bands included in figure 6g
Vinculin

## Slide 20
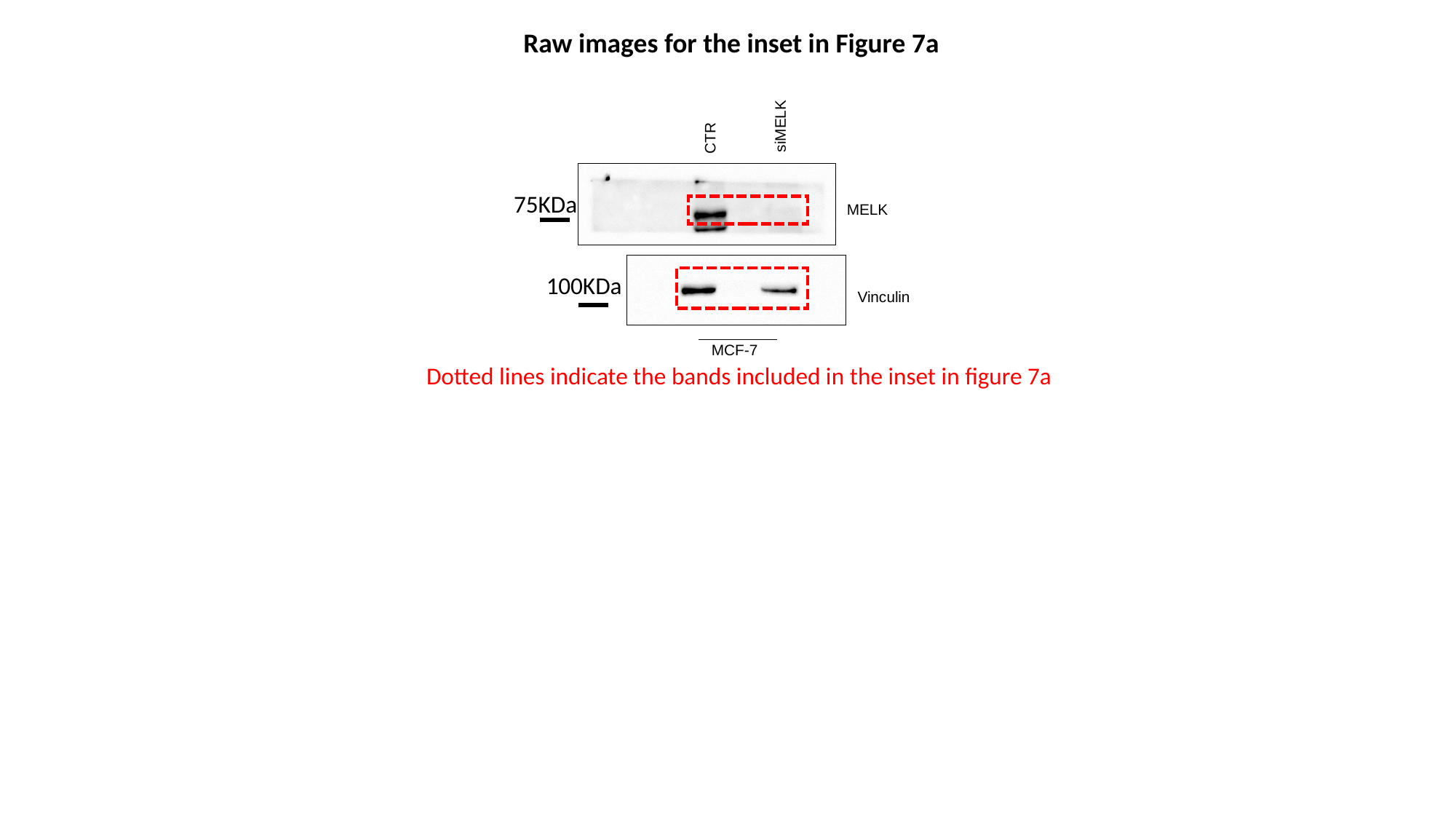

Raw images for the inset in Figure 7a
siMELK
CTR
MELK
Vinculin
MCF-7
75KDa
100KDa
Dotted lines indicate the bands included in the inset in figure 7a
