# Supplementary material for: Selective impact of ALK and MELK inhibition on ERα stability and cell proliferation in cell lines representing distinct molecular phenotypes of breast cancer
Source: Sci Rep. 2024 Apr 8;14:8200. doi: 10.1038/s41598-024-59001-x (PMC11001865; doi:10.1038/s41598-024-59001-x)
Supplement: Supplementary file 9 — Supplementary Legends. [file 41598_2024_59001_MOESM9_ESM.docx]

**Supplementary Figure Captions.**

**Figure 1. Correlation between two ALK inhibitors.**

Linear regression and Spearman Correlation values for the effective concentration 50 (EC_50_) for inhibitor-induced reduction in ERα intracellular levels between the ALK inhibitors AZD3436 – AZD and and AP26113 – AP in all the seven BC cell lines used in the study. Main panels display the correlation coefficient (r) and p-values.

**Figure 2. Original Western blots.**

Raw images of all the replicates for each experiment are indicated using the figure numbering as in the main text. Blots have been performed as indicated in the Material and Method Section of the main text.
